# Chemistry

**Published:** 1837-01

**Authors:** 


### CHEMISTRY.

*New Method of preparing Iodic Acid.* By Lewis Thompson, Esq. M.R.C.S.

This method is said "to be cheaper and safer than the mode used by Sir H. Davy, and affords a purer acid than the plan proposed by Gay Lussac;" it is described as follows: "Put one atom, or 124 grains, of iodine into a bottle containing twenty-four ounces of water, and pass chlorine, previously washed, through the mixture, until this, having undergone various changes of colour, from orange-red to yellow, shall have become as colourless and transparent as water. The solution is then to be carefully heated to 212° Fahr., to expel the excess of chlorine, and five atoms, or 690 grains, of pure oxide of silver being added, the mixture is to be boiled for ten minutes, filtered, and cautiously evaporated to dryness. The product is pure iodic acid, and must be kept in a well-stoppered phial."

*Medical Gazette, November* 5, 1836.

